# Unravelling the Enigma of Ocular Complexity: Delving into Aniridia, Xerophthalmia, Corneal Ulcer, Keratomalacia, and Beyond

**DOI:** 10.7759/cureus.64631

**Published:** 2024-07-16

**Authors:** Renu Magdum, Rutu K Rao, Aditya Ganesh, Neha Chaudhary, Vishakha Vatkar

**Affiliations:** 1 Ophthalmology, Dr. D. Y. Patil Medical College, Hospital and Research Centre, Pune, IND

**Keywords:** visual deterioration, ocular complexity, corneal ulcer, xerophthalmia, aniridia

## Abstract

In this case report, we explore a complex and rare presentation of simultaneous aniridia, xerophthalmia, corneal ulcer, and keratomalacia in a 21-year-old patient with a congenital history of nystagmus and progressively diminishing visual acuity. The patient experienced a recent exacerbation of ocular pain and redness, primarily in the right eye, which led to a comprehensive ophthalmological evaluation. Examination revealed corneal ulceration, vascularization, and the absence of the iris, posing a unique clinical challenge. In this report, we detail the personalized management and therapeutic strategies employed in this case, emphasising the need for an integrated and holistic approach to treating complex ocular conditions. The outcomes highlight the importance of tailored treatment plans and a thorough understanding of the interplay between various eye pathologies to achieve better patient outcomes.

## Introduction

Aniridia is an uncommon genetic eye condition in which the iris, the colored part of the eye, is absent. This absence can affect vision and is commonly linked with other eye conditions such as cataracts, glaucoma, and underdevelopment of the fovea, which is crucial for sharp central vision [1]. In individuals with aniridia, the deficiency of a functional iris can worsen the impact of xerophthalmia. Without a proper iris, the eye has difficulty controlling the amount of light entering it and cannot properly shield against injuries, raising the risk of further eye complications. Apart from the problem of light regulation, aniridia can disrupt the overall stability of the eye's environment.

Xerophthalmia is a serious eye disorder marked by extreme dryness and can lead to keratomalacia and corneal ulcers. This condition poses a significant health concern, particularly in developing countries where vitamin A deficiency is prevalent. Xerophthalmia is a progressive eye disease caused by inadequate vitamin A intake, leading to the drying and hardening of the eye's surface layers. This deficiency can result in several eye problems, such as conjunctival and corneal xerosis, Bitot's spots, keratomalacia, nyctalopia, and retinopathy [2]. It also increases the risk of eve infections and can ultimately cause total blindness if not properly addressed.

Additionally, aniridia especially affects young children. Nutritional deficiency in a developing country is common. Such deficiency can cause xerophthalmia and lead to blindness, limit growth, weaken innate and acquired host defences, and exacerbate infection [3]. Bitot's spots, which are foamy, white patches on the conjunctiva, are a telltale sign of severe vitamin A deficiency. Keratomalacia, involving the softening and potential liquefaction of the cornea, can lead to the formation of ulcers and scars.

Dealing with aniridia, xerophthalmia, keratomalacia, and corneal ulcers together presents a particularly severe and complex clinical scenario. It is crucial to understand the symptoms and potential consequences of these conditions for timely diagnosis and effective management. Taking a comprehensive approach is essential to prevent permanent vision loss and enhance the patient's quality of life. This involves addressing nutritional deficiencies, providing appropriate medical treatments, and, when necessary, considering surgical interventions. By doing so, we can significantly improve the prognosis for these patients and help them maintain better overall eye health.

In this report, we aimed to provide a comprehensive analysis of aniridia, especially when combined with xerophthalmia, keratomalacia, and corneal ulcer.

## Case presentation

The patient, a 21-year-old individual, presented with a history of progressive vision loss and ocular discomfort. The chief complaint was a significant diminution of vision in the right eye, which started insidiously a year ago and gradually worsened. The left eye also experienced a similar decline in vision, beginning three to four months after the right eye. Additionally, the patient reported ocular pain in the right eye for the past two days, accompanied by redness. 

The patient had a congenital history of nystagmus and gave a history of using spectacles for four to five years for diminution of vision, which they discontinued approximately five years ago. There was no history of photophobia, double vision, flashes, floaters, or ocular trauma. The patient's past medical history was notable for visits to a hospital during childhood for diminution of vision and nystagmus. There was no history of systemic illnesses such as diabetes, hypertension, tuberculosis, bronchial asthma, ischemic heart disease, epilepsy, or major surgeries. The patient had no known allergies to medications or food. Birth history indicated a home birth without medical supervision, and there was a family history of blindness, with the father being blind since birth. A relative of the patient reported difficulty in understanding and performing tasks compared to peers, suggesting mild intellectual disability.

The patient did not complain of abdominal pain, blood in the urine, fever, burning micturition, or difficulty in walking. Upon physical examination, the patient was conscious, oriented, and cooperative. The patient was vitally stable and weighed about 54 kg. A review of systems revealed no significant findings in cardiovascular, respiratory, gastrointestinal, or genitourinary systems.

The ocular examination revealed significant findings. We noted visual acuity in the right eye as no perception of light. The left eye had slightly better vision (perception of light was present) but was still severely impaired. On examination, inflammation of both eyelids in both eyes was characterized by oily secretion along the meibomian gland suggestive of meibomian gland dysfunction. On further evaluation, which we conducted with the Schirmer test 1, the results were 5 mm for the right eye and 7 mm for the left eye, indicating severe dryness. Results for the Schirmer test 2 were 20 mm for the right eye and 25 mm for the left eye. 

**Figure 1:**
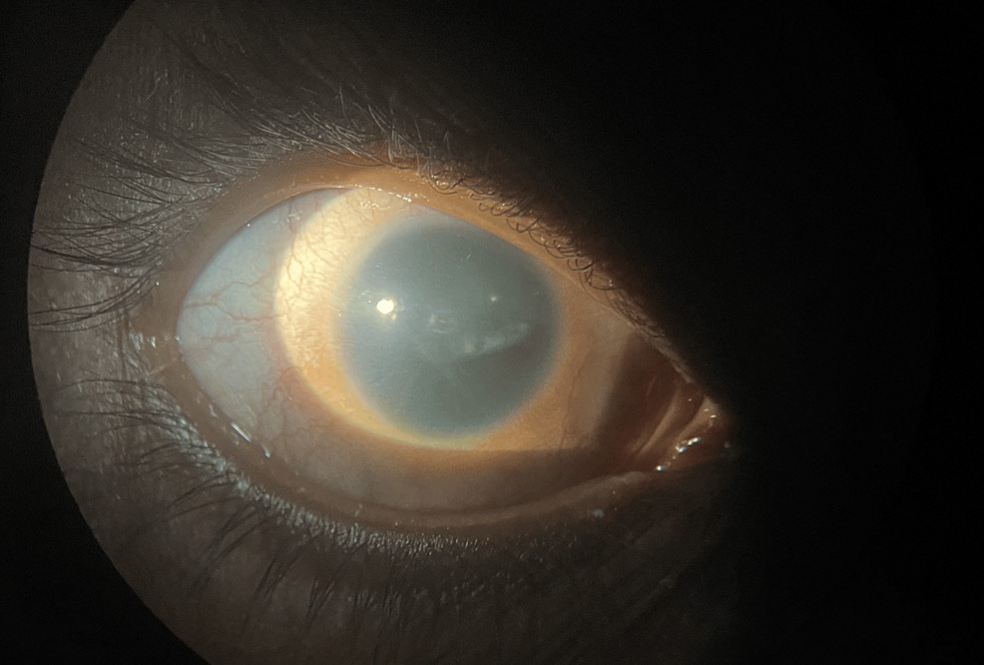
Anterior segment of right eye in diffuse illumination

**Figure 2:**
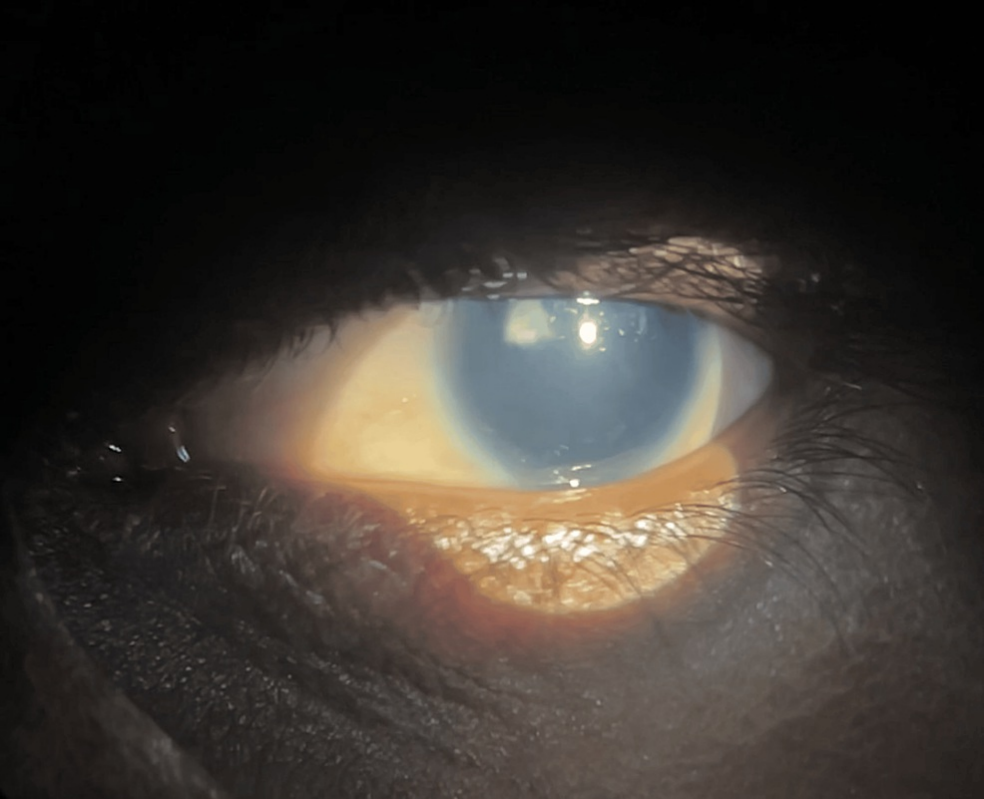
Anterior segment of left eye in diffuse illumination

The examination also showed conjunctival xerosis with Bitot's spots at seven o'clock and a 1x1 mm central corneal ulcer in the right eye. There was superficial vascularization and 360-degree increased curvature of the cornea. Upon further detailed examination, we suspected limbal stem cell deficiency because corneal conjunctivalization was present and corneal clarity was lost. We also noted stippling staining on fluorescein staining. On fundus examination, both eyes exhibited complete aniridia, reduced corneal sensation, and optic atrophy. The lens showed subluxation superiorly in the right eye (Figure 1) and posterior subcapsular cataract (PSC) in the left eye (Figure 2).

Diagnostic tests included a B-scan ultrasonography, which indicated intact posterior chambers and vitreous degeneration in both eyes. The retina appeared well attached in both eyes. The blood tests showed no such abnormalities.

The patient was started on a comprehensive medical treatment regimen. This included oral ascorbic acid (1000 mg/day), multivitamin capsules, and topical treatments such as homatropine 2% eye drops twice a day, moxifloxacin 0.5% eye drops four times a day, and hourly lubricating eye drops. We provided vitamin A supplementation in high doses (2,00,000 IU). Additionally, travoprost plus timolol eye drops and brimonidine plus brinzolamide eye drops twice a day were included to manage intraocular pressure. 

After following the treatment, the patient showed a reduction in redness, pain, and superficial vascularization. However, visual improvement was limited owing to underlying structural abnormalities such as optic atrophy and aniridia. We discharged the patient with instructions for continued use of medications and advised them to seek immediate medical attention if symptoms worsened. The present case underscores the complexity of managing concurrent ocular conditions and highlights the need for a multidisciplinary approach to treatment and care.

## Discussion

This case involves a 21-year-old patient with a complex history of eye problems, making it particularly challenging to manage properly. The patient presented with congenital nystagmus and aniridia with a gradual decline in vision owing to xerophthalmia and corneal ulcers. The Schirmer test results show severe dry eye disease, which adds significant difficulty in managing the health of the cornea and maintaining vision.

The distribution of the Schirmer test results indicated altered tear film dynamics and potential tear film instability, as evidenced by the variations in tear break-up time between the right and left eyes. Tear film abnormalities can contribute to ocular surface irregularities and visual disturbances, emphasizing the importance of tear film assessment in the evaluation of ocular surface health [4]. Moreover, the major disparity in the Schirmer tests 1 and 2 showed that the patient’s baseline tear production was significantly impaired (dry eye condition); the lacrimal glands were still functional and could produce tears adequately under stimulation. This suggested that the dry eye condition could be managed with treatments aimed at increasing baseline tear production and reducing tear evaporation [5].

The ocular examination showed abnormalities suggesting an irregular corneal surface. This raised concerns about potential conditions such as keratoconus or irregular astigmatism, both of which can significantly affect visual acuity and quality of life [6]. To understand better the extent and severity of these corneal irregularities, we recommend further imaging studies, such as corneal tomography. These studies will help guide appropriate management strategies, which might include specialty contact lenses or corneal refractive surgery [7].

Additionally, the presence of aniridia and lens abnormalities such as subluxation and posterior subcapsular cataracts further complicated the clinical picture, potentially contributing to visual impairment and discomfort. The conjunctival congestion and xerosis, along with Bitot’s spots observed at seven o’clock, indicated ocular surface inflammation and vitamin A deficiency, respectively. Moreover, the corneal examination revealed significant abnormalities, including superficial vascularization and increased curvature, indicative of corneal pathology. The presence of a corneal ulcer in the right eye, coupled with an irregular corneal surface, underscored the severity of the ocular condition.

In the fundus examination, the media in the right eye appeared hazy, possibly owing to underlying pathology or poor media clarity. We noted optic atrophy in the right eye, indicating damage to the optic nerve and potential vision loss. The presence of optic disc abnormalities underscored the need for further evaluation to determine the underlying etiology and assess the extent of optic nerve damage. On examination of the anterior segment, several notable findings emerged. The presence of nystagmus in the right eye indicated an involuntary rhythmic movement of the eye, which may have contributed to visual impairment. Reduced corneal sensation in both eyes suggested compromised corneal integrity, potentially contributing to decreased protective reflexes and predisposing the cornea to injury or infection.

Managing this case required a multidisciplinary approach to anticipate aniridia, xerophthalmia, and corneal ulcers. The current medication covers critical aspects but further evaluation to explore other medication approaches is necessary to enhance medical treatment outcomes. To manage xerophthalmia, the current medication was vitamin A tablets (2,00,000 IU). This high dose of vitamin A is essential for treating xerophthalmia, which is caused by vitamin A deficiency [8]. The regimen correctly included this critical component. For better effectiveness of the treatment plan, long-term adequate levels of vitamin A are necessary, and from different dietary sources.

Moreover, the inflammation of the eyelids and meibomian glands, noted as 'eyelid meibomitis' and ‘eyelash blepharitis’, highlights the need to focus on ocular surface health and maintaining tear film stability. Chronic inflammation in these areas can cause evaporative dry eye syndrome, affecting both comfort and visual function [9]. To address these issues, targeted management strategies are crucial. These may include practices such as lid hygiene, warm compresses, and lubricating eye drops to relieve symptoms and enhance ocular surface health [10].

Furthermore, some researchers suggest the association between limbal stem cell deficiency and aniridia. They consider mutation in the *PAX6* gene to be the primary etiology concerned with both limbal stem cell deficiency and aniridia [11]. During the development of new tissues and organs, it is believed that the *PAX6* gene plays an important role. Therefore, a mutation in the *PAX6* gene can lead to various pathologies such as aniridia, peters anomaly, coloboma, microphthalmia, and WAGR (Wilms tumor, aniridia, genitourinary anomalies, and (mental) retardation) syndrome.

In addition, the patient can develop glaucoma as there is a chance of anterior rotation of rudimentary iris during the course and peripheral anterior synechiae can also occur. To prevent further complications such as glaucoma, we started the patient on an anti-glaucoma drug. However, medications usually fail to control glaucoma and surgical intervention is necessary. Glaucoma presenting before one year of age is difficult to treat. Surgery is often necessary for glaucoma by age 20 in patients with congenital aniridia. The options for surgical therapy include goniotomy, trabeculotomy, trabeculectomy with mitomycin C, and combined trabeculectomy and trabeculotomy. Glaucoma drainage devices may be implanted as a primary modality in aniridic glaucoma [12].

## Conclusions

The comprehensive evaluation of the patient's ocular health revealed a complex interplay of pathologies involving the anterior segment and fundus. The presence of nystagmus, reduced corneal sensation, conjunctival xerosis, corneal ulceration, aniridia, and optic atrophy highlights the severity and multifactorial nature of the ocular condition. These findings underscore the importance of a multidisciplinary approach to management, incorporating ophthalmological, neurological, and systemic evaluations. Addressing the patient’s ocular discomfort and visual impairment requires targeted interventions tailored to the specific pathologies identified.

Management strategies may include topical medications to promote corneal healing, vitamin supplementation to address nutritional deficiencies, and interventions to manage underlying systemic conditions contributing to ocular pathology. Additionally, optimizing ocular surface health through lid hygiene measures, lubricating eye drops, and anti-inflammatory treatments are essential for maintaining ocular comfort and preventing disease progression.
